# Hypocholesterolemic and Hepatoprotective Effects of “Triguero” Asparagus from Andalusia in Rats Fed a High Cholesterol Diet

**DOI:** 10.1155/2012/814752

**Published:** 2011-11-17

**Authors:** M. D. García, R. De la Puerta, M. T. Sáenz, A. Marquez-Martín, M. A. Fernández-Arche

**Affiliations:** ^1^Department of Pharmacology, Faculty of Pharmacy, University of Seville, C. Professor García Gonzalez 2, 41012 Seville, Spain; ^2^Department of Pharmacology, Therapeutics and Toxicology, Institute of Neurosciences, Faculty of Medicine, Universitat Autònoma de Barcelona, 08193 Bellaterra, Spain

## Abstract

The cultivated species of the wild autochthonous *Asparagus officinalis* in Andalusia in Spain is commonly called “triguero” asparagus. This vegetable has traditionally been very much appreciated for its organoleptic and nutritional characteristics. This study has been designed to evaluate the potential effect of different concentrations of freeze-dried asparagus (500, 250, and 125 mg/Kg of body weight/day) on oxidative status and lipid profile in rats fed a cholesterol-rich diet. After five weeks of treatment, doses of 250 and 500 mg/Kg of asparagus were able to significantly reduce total cholesterol and LDL cholesterol levels. Atherogenic index was also significantly reduced in a dose-dependent manner by administrating freeze-dried asparagus. A beneficial effect was observed in the HDL cholesterol levels in asparagus-fed groups although the increase was not significant. Consumption of asparagus also improved antioxidant status, assayed superoxide dismutase (SOD) and catalase (CAT) enzymes, and protected against lipid peroxidation. These results show that the intake of green asparagus from Andalusia (Spain) helps to regulate plasma lipid levels and prevents oxidative damage in hypercholesterolemic conditions.

## 1. Introduction

Atherosclerosis is known to be the principal contributor to the pathogenesis of myocardial and cerebral infarctions, and these are currently the leading cause of morbidity and mortality worldwide [1]. Hypercholesterolemia, or more specifically elevated plasma total cholesterol and low-density lipoprotein cholesterol (LDL-c), is an important risk factor for the development and progression of atherosclerosis [2]. Diet is the first therapeutic approach to treat hyperlipidemia. Combination of antioxidants and hypocholesterolemic agents in diets is crucial to upset cholesterol levels and hence to restrict the development of atherosclerotic lesions. A number of vegetables with potential bioactive components, such as polyphenols, sterols, and fibre, have been investigated for their antihyperlipidemic, antioxidant, and antiatherosclerotic properties [3, 4]. Some studies have investigated the cholesterol-lowering properties and hepatoprotective effect of asparagus from different species and world areas, such as *Asparagus racemosus* from India [5] or *Asparagus officinalis* by-products from China [6].

“Triguero” asparagus from Andalusia (Spain) is a product traditionally used in Spain, very appreciated for its organoleptic and nutritional characteristics. Although this species contains an interesting composition in bioactive components, it's functional properties have been poorly studied. It has been established that, among the most commonly consumed vegetables, green asparagus from Andalusia presents higher antioxidant capacity [7] than other asparagus from other world areas, and this property has been associated to a great extent to its total phenolic content [8]. It has been demonstrated that the flavonoid rutin represents 60–80% of the total phenolic content of purple and green asparagus extracts and that content of rutin could be partially responsible for its antioxidant properties [9].

The aim of the current study was to evaluate the hypocholesterolemic effect of “triguero” asparagus from Andalusia by analyzing its effects on plasma lipid levels and its influence on the hepatic antioxidant status of rats fed a cholesterol-rich diet.

## 2. Subjects and Methods

### 2.1. Plant Material

Cultivated “triguero” asparagus comes from wild asparagus autochthonous (*Asparagus officinalis*) from the Huétor-Tájar area (Andalusia, Spain). On harvest day, asparagus spears were transported to the laboratory and then weighed, frozen at −20°C, and freeze-dried. This plant tissue was ground into a fine powder and stored at −20°C for the experiments.

### 2.2. Animals and Diets

Thirty male Wistar rats, weighing 100–125 g, purchased from Central Animal House of Espartinas (Seville, Spain) were housed in an air-conditioned room at 25 ± 1°C and 65–70% relative humidity with a 12 h light-dark cycle. The protocol used in this study was approved by the Ethic Committee for Animal Experimentation of the University of Seville (Spain), based on the recommendations of the European Council (86/609/EEC). Diets were made following American Institute of Nutrition (AIN) recommendations [10]. Animals were randomly assigned to five groups (*n* = 6). The first group was fed a standard diet (Harlan S. L), called SD. The second group was fed the standard diet supplemented with 1% cholesterol and 0.20% cholic acid, called HCD. Asparagus-treated groups, received standard diet supplemented with 1% cholesterol, 0.20% cholic acid, and freeze-dried asparagus at doses of 125, 250, and 500 mg/kg of animal, respectively, during 5 weeks. These last were named FA-1, FA-2, and FA-3. The animals were given food and water *ad libitum* during the experimental period. Diet intake and the body weight were weekly recorded.

### 2.3. Tissue Preparations

At the end of the treatment, the animals were kept for 24 h fasting, and rats in all groups were anaesthetized; their blood was drawn by cardiac puncture and heparinised. Blood samples were centrifuged at a speed of 4000 g (4 min, 4°C) to obtain the plasma.

The livers were collected, weighed, and latter rinsed with physiological saline. All samples were stored at −80°C until analyzed.

### 2.4. Plasma Lipid Profiles and Atherogenic Index (AI) Values

Concentrations of total cholesterol (TC), triglycerides (TG), and cholesterol high-density lipoprotein (HDL-c) in plasma were determined by enzymatic colorimetric methods, using commercial kits (Spinreact). Cholesterol low-density lipoprotein (LDL-c) was accomplished according to the procedures described by Friedewald et al. [11].The atherosclerosis index (AI) was defined as (TC−HDL-c)/HDL-c) and was calculated for the experimental groups.

### 2.5. Evaluation of Antioxidant Status

Liver homogenates (10% w/v) were prepared in 0.25 M sucrose, 1 mM EDTA, 1 mM DL-dithiothreitol, and 15 mM Tris-HCl (pH 7.4). Each homogenate was centrifuged at 800 g for 20 min at 4°. The supernatant was used to determine hepatic enzyme activities. Catalase activity (CAT) was measured by the method described by Aebi [12]. Changes in absorbance were recorded at 240 nm. CAT activity was calculated in terms of mU/mg protein. Superoxide dismutase (SOD) activity was estimated using the xanthine-oxidase-cytochrome C method [13]. The inhibition of xanthine-oxidase was followed spectrophotometrically at 550 nm. The activity was expressed in U/mg of protein. Lipid peroxidation was estimated according to the method of Esterbauer and Cheeseman [14]. Concentrations were determined from a standard curve by using 1,1,3,3- tetraethoxypropane and was expressed as nmol of malondialdehyde (MDA) formed per 100 mg of tissue.

### 2.6. Statistical Analysis

All data presented are the mean ± standard errors from three measurements. Statistical differences were calculated using “Anova test”, followed by “Dunnet Test”. Differences were considered significant at *P* < 0.05.

## 3. Results

### 3.1. Animal Weights and Food Intake

**Figure 1:**
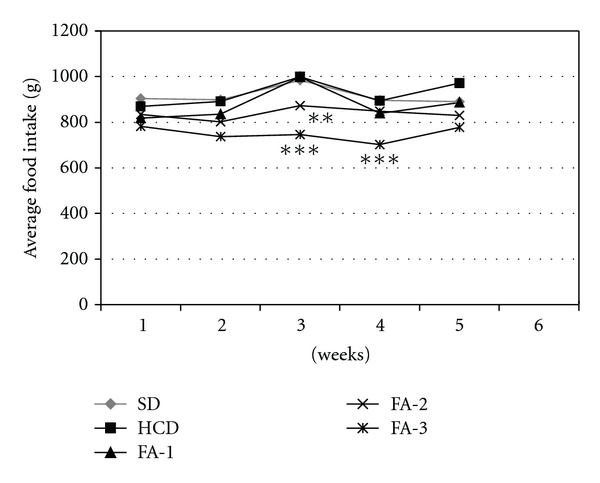
Average weekly food intake (g) of rats from SD, HCD and HCD supplemented with freeze-dried asparagus: 125 mg/kg (FA-1); 250 mg/kg (FA-2); 500 mg/kg (FA-3) groups, during 5-week feeding period. Each bar represents mean ± SE from six rats. **P < 0.01; ***P < 0.001.

**Figure 2:**
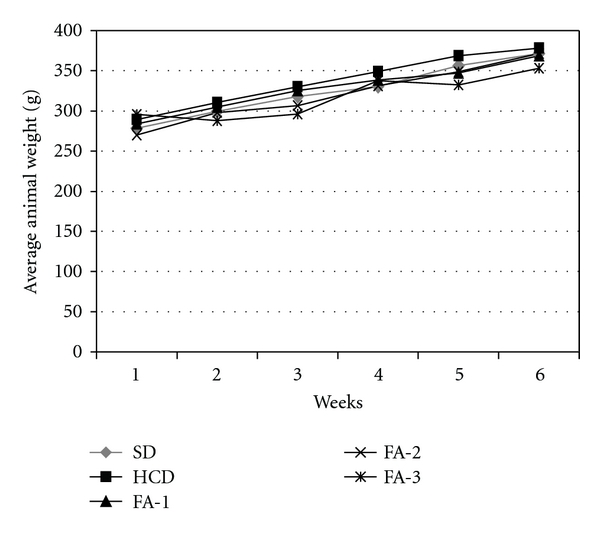
Average animal weight (g) of rats from SD or HCD and HCD supplemented with freeze-dried asparagus: 125 mg/kg (FA-1); 250 mg/kg (FA-2); 500 mg/kg (FA-3) groups, during 5-week feeding period. Each bar represents mean ± SE from six rats.

The weekly mean food intake of the rats during the feeding period is showed in Figure 1. There was a significant reduction in the food intake in the groups treated with the highest and intermediate asparagus doses in respect to the hypercholesterolemic group. It was also observed a tendency to reduce the body weight in the asparagus-fed groups although the differences did not reach significant values (Figure 2).

### 3.2. Plasma Lipid Profile and AtherogenicIndex (AI)

**Figure 3:**
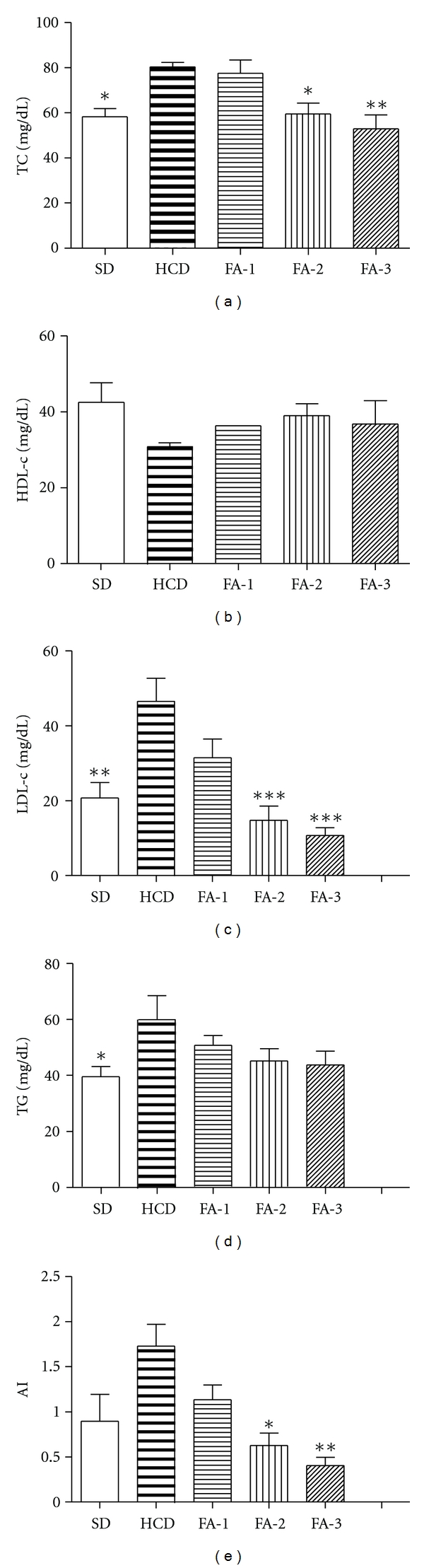
Changes in plasma lipid levels of rats from SD, HCD, and HCD supplemented with freeze-dried asparagus: 125 mg/kg (FA-1); 250 mg/kg (FA-2); 500 mg/kg (FA-3) groups. (a) Effects in TC; (b) effects in HDL-c; (c) effects in LDL-c; (d) effects in TG; (e) Effects in atherogenic index: (TC)−(HDL-c)/HDL-c. Each value represents mean ± SE from six rats. *P < 0.05; **P < 0.01; ***P < 0.001 versus HCD group.

Figure 3 shows the plasma lipid levels at the end of the experiment. After five weeks of feeding, the plasmatic TC, TG, and LDL-c concentrations of rats from HCD group showed a significant increase compared with those of rats from SD group (*P* < 0.05; *P* < 0.01), and a decrease of HDL-c concentration in HCD group was also observed. Freeze-dried asparagus (FA) was able to significantly reduce total cholesterol and LDL-c levels (*P* < 0.01, *P* < 0.05, and *P* < 0.001) at doses of 500 and 250 mg. In the HDL-c and TG levels, a beneficial effect was observed in the asparagus treated groups, although these effects were not significant. AI was reduced in a dose-dependent manner by the administration of FA, being significant at 250 and 500 mg/Kg doses (*P* < 0.05, *P* < 0.01). 

### 3.3. Changes in Liver Weights

**Figure 4:**
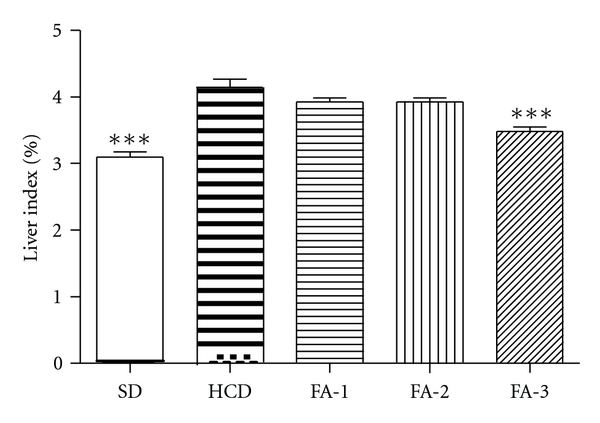
Effects in liver index of rats from SD, HCD and HCD supplemented with freeze-dried asparagus: 125 mg/kg (FA-1); 250 mg/kg (FA-2); 500 mg/kg (FA-3) groups. Liver index = liver weight (g)/body weight (g) ×100. Each bar represents mean ± SE from six rats. ***P < 0.001 versus HCD group.

Figure 4 shows the induced changes in the liver weights in rats fed with cholesterol-enriched diet compared to cholesterol-enriched and supplemented with FA. As it can be seen in Figure 4, FA treatments were able to reduce the liver index (LI) or the weight of this organ in respect to body weight. This reduction was statistically significant at the higher dose (*P* < 0.001).

### 3.4. Antioxidant Status

**Figure 5:**
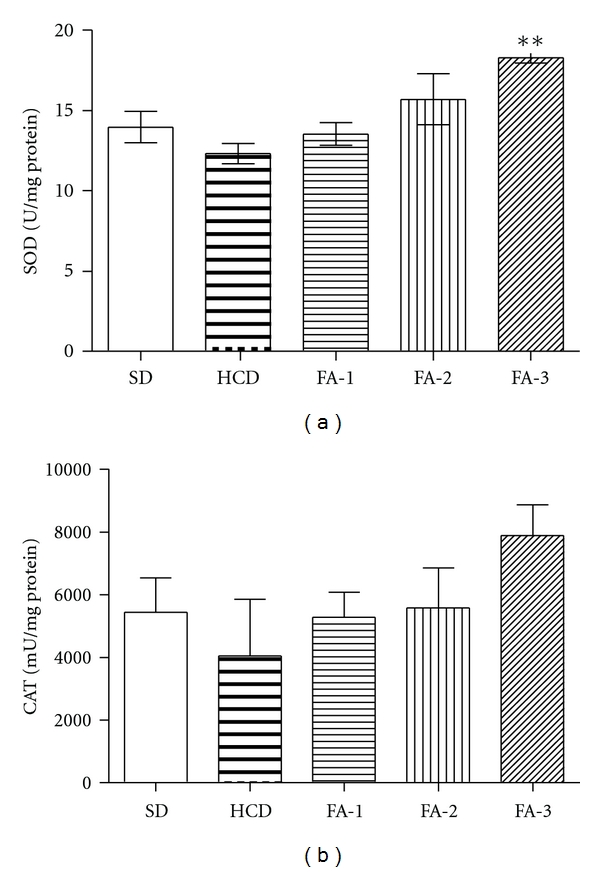
Effects on antioxidant enzymes of livers of rats from SD, HCD, and HCD supplemented with freeze-dried asparagus: 125 mg/kg (FA-1); 250 mg/kg (FA-2); 500 mg/kg (FA-3) groups. (a) Superoxide dismutase activity (SOD); (b) Catalase activity (CAT). Each bar represents mean ± SE from six rats. **P < 0.01 versus HCD group.

**Figure 6:**
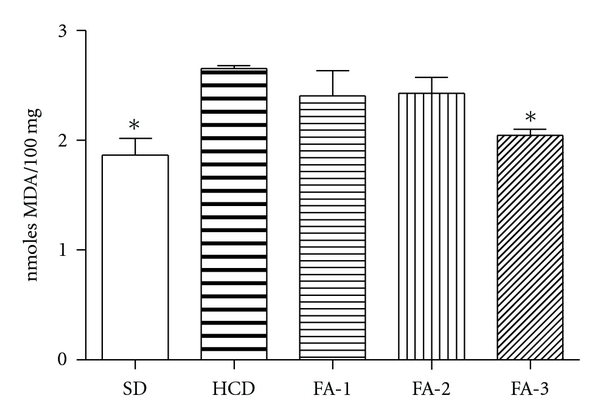
Effects on lipid peroxidation of livers of rats from SD, HCD, and HCD supplemented with freeze-dried asparagus: 125 mg/kg (FA-1); 250 mg/kg (FA-2); 500 mg/kg (FA-3). Each bar represents mean ± SE from six rats. *P < 0.05; versus HCD group.

SOD and CAT activities in the livers are illustrated in Figure 5. The high dose of FA prevents the reduction of both enzyme activities, SOD and CAT induced by cholesterol rich diet, although only SOD activity was significatively increased. As shown Figure 6, when MDA levels of liver from rats HCD group were compared with those obtained from SD group, a significant increase was observed (*P* < 0.05) in this last group. Rats treated with the highest dose of FA showed a significant decrease (*P* < 0.05) in the concentration of MDA measured in liver tissues compared to those from hypercholesterolemic rats group.

## 4. Discussion

The present study was designed to evaluate the effects of a diet supplemented with green freeze-dried asparagus (FA) from Andalusia (Spain) on plasma lipid levels and hepatic antioxidant enzyme activities in hypercholesterolemic rats. Rats fed a diet rich in cholesterol showed increased plasmatic levels of TG, TC, and LDLc and decreased circulating HDLc, thus providing a model for dietary hyperlipidemia studies [15]. Epidemiological studies indicate that supplemental dietary fibre protects against the development of overweight, which is an important cardiovascular risk factor widely associated to high fat diets [16]. Our results showed a food intake reduction in the group of animals treated with the higher asparagus dose. This finding could be attributed to the high-fibre content of this vegetable [17].

An increased concentration of serum cholesterol increases the risk of developing cardiovascular disease [18]. After treatment with FA for a period of 5 weeks, a significant decrease in plasmatic TC accompanied by a reduction in its LDLc fraction was observed in hyperlipidemic rats. LDLc is a major risk factor in cardiovascular disease and is also the target of many hypocholesterolaemic therapies. FA also showed a beneficial effect, causing a weak increase in HDL-c level, although the differences were not statistically significant. Similarly FA slightly decreased TG levels compared to HCD group. Atherogenic index (AI) is considered to be an important parameter of atherosclerosis. We found that HCD group suffered a significant rise in AI compared to animals from SD group, whereas the animals treated with FA (250 and 500 mg/Kg) presented a decrease in this value. These data indicates that the FA highest doses could effectively improve the plasmatic lipid profile. In this context, the presence of bioactive components in “triguero asparagus,” such as phytosterols, saponins, and fibre, may play a role in cholesterol reduction, [16, 19, 20]. On the other hand, it is well documented that high-cholesterol-rich diet can cause liver damage by oxidation [21]. When rats are fed cholesterol-rich diet, their livers, the primary organ that metabolizes cholesterol ingested in excess, is affected by oxidative stress which leads to lipid peroxidation. In this study, oral administration of FA prevented the cholesterol-rich-diet-induced MDA elevation. The treatment also resulted in a significant decrease of liver MDA with the highest doses of FA, suggesting that FA might be capable of attenuating or slowing down oxidative stress-related lipid peroxidation.

It has been published that during oxidative stress tissues respond by the induction of antioxidant mechanism [22]. However, in an excessive oxidative stress situation, the upregulation of defense system could not protect completely against the oxidative damage produced by free radicals. In the present study, we have observed decreased activities of antioxidant enzymes SOD and CAT in the liver of rats fed on HCD as compared to those on standard diet. Our results are in agreement with reports of other workers, which suggest that feeding an HCD to experimental animals depresses their antioxidant system due to increased lipid peroxidation and formation of free radicals [23]. The present study showed that simultaneous treatment of cholesterol-fed rats with doses of FA improved antioxidant defense, increasing SOD and CAT activities. SOD activity was significantly increased at the highest dose. These effects could be attributed to a protective antioxidant effect produced by the high content of flavonoids and other phenolic compounds of “triguero” asparagus, as it has already been described for other flavonoid-rich plants [24].

In summary, this study shows that diet supplementation with “triguero” asparagus is able to prevent atherogenic risk markers as well as to prevent the oxidative hepatic damage in hypercholesterolemic conditions. Likely, functional components present in this asparagus variety, as flavonoids and steroidal saponins, could be responsible, at least in part, for this protective effect.
